# Supplementary material for: Comparative genomics reveals high genetic similarity among strains of Salmonella enterica serovar Infantis isolated from multiple sources in Brazil
Source: PeerJ. 2024 May 20;12:e17306. doi: 10.7717/peerj.17306 (PMC11114117; doi:10.7717/peerj.17306)
Supplement: Supplemental Information 2 [file peerj-12-17306-s002.pdf]

Table S1 – Occurrence of prophages in the 80 Brazilian genomes of *Salmonella* Infantis strains analyzed in the present study.

Strain	Year	Isolation source	Isolation material	Prophages
SI 1348/13	2013	Human	Human feces	Gifsy_1
SI 2385/13	2013	Food	Soy	Yersin_L_413C
SI 2950/13	2013	Human	Human feces	Salmon_Fels_1, Yersin_L_413C
SI 2951/13	2013	Human	Human feces	Gifsy_1, Escher_pro483
SI 3156/13	2013	Environment	Disposable shoe cover	Entero_BP_4795
SI 5025/13	2013	Human	Human feces	Gifsy_1, Yersin_L_413C, Salmon_SPN3UB, Entero_P4
SI 124/14	2014	Animal	Swine feces	Salmon_Fels_1, Entero_ES18
SI 210/14	2014	Environment	Dragging swab	Gifsy_1
SI 212/14	2014	Environment	Dragging swab	Yersin_L_413C
SI 388/14	2014	Animal feed	Soybean animal meal	Salmon_Fels_1, Entero_BP_4795
SI 583/14	2014	Food	Chicken carcass	Gifsy_1, Entero_BP_4795, Salmon_118970_sal3
SI 584/14	2014	Food	Pasta containing ham	Gifsy_1, Entero_BP_4795, Salmon_118970_sal3
SI 677/14	2014	Food	Carcass cleaning wipe	Salmon_Fels_1, Stx2_c_1717
SI 723/14	2014	Environment	Dragging swab	Salmon_Fels_1, Entero_BP_4795, Salmon_118970_sal3
SI 982/14	2014	Animal	Chicken feces	Gifsy_1
SI 1143/14	2014	Animal	Chicken feces	Salmon_Fels_1, Salmon_vB_SosS_Oslo
SI 1284/14	2014	Environment	Dragging swab	Salmon_Fels_1, Entero_BP_4795, Salmon_118970_sal3
SI 1380/14	2014	Animal	Chicken feces	Gifsy_1, Salmon_vB_SosS_Oslo
SI 1408/14	2014	Human	Human feces	Gifsy_1, Escher_pro483
SI 1409/14	2014	Human	Human feces	Gifsy_1, Yersin_L_413C
SI 1441/14	2014	Food	Mayonnaise	Salmon_Fels_1, Yersin_L_413C, Entero_YYZ_2008
SI 1711/14	2014	Animal	Chicken feces	Gifsy_1, Escher_pro483
SI 2378/14	2014	Environment	Truck swab	Gifsy_1, Entero_BP_4795, Salmon_118970_sal3
SI 2430/14	2014	Food	Mixed meat sausage	Gifsy_1, Entero_BP_4795, Salmon_118970_sal3
SI 2461/14	2014	Food	Chicken carcass	Entero_BP_4795, Salmon_118970_sal3
SI 2463/14	2014	Food	Chicken carcass	Salmon_Fels_1, Entero_BP_4795, Salmon_118970_sal3
SI 2548/14	2014	Animal	Chicken feces	Salmon_Fels_1, Escher_pro483
SI 3836/14	2014	Environment	Dragging swab	Salmon_Fels_1, Escher_pro483
SI 4882/14	2014	Food	Chicken carcass	Salmon_Fels_1, Salmon_SPN3UB

Strain	Year	Isolation source	Isolation material	Prophages
SI 4892/14	2014	Food	Chicken wings	Salmon_Fels_1, Salmon_SPN3UB
SI 4895/14	2014	Food	Chicken carcass	Gifsy_1, Salmon_SPN3UB
SI 4901/14	2014	Food	Pig snout	Salmon_Fels_1, Salmon_SPN3UB
SI 5247/14	2014	Food	Chicken upper leg and thigh	Gifsy_1, Salmon_SPN3UB
SI 342/15	2015	Food	Swine heart	Salmon_Fels_1, Entero_BP_4795, Salmon_118970_sal3
SI 444/15	2015	Food	Pork filet	Gifsy_1, Entero_BP_4795, Salmon_118970_sal3
SI 447/15	2015	Food	Smoked and salted pork meat	Gifsy_1, Entero_BP_4795, Salmon_118970_sal3
SI 1809/15	2015	Animal feed	Meat animal meal	Gifsy_1, Salmon_118970_sal3
SI 1816/15	2015	Animal feed	Poultry viscera animal meal	Salmon_Fels_1, Entero_BP_4795, Salmon_118970_sal3
SI 2280/15	2015	Food	Chicken carcass	Gifsy_1, Entero_BP_4795, Salmon_118970_sal3
SI 2302/15	2015	Environment	Cleaning wipe	Salmon_Fels_1, Yersin_L_413C
SI 2370/15	2015	Food	Carcass cleaning wipe	Salmon_Fels_1, Yersin_L_413C
SI 2869/15	2015	Food	Chicken upper leg	Salmon_Fels_1, Salmon_SPN3UB
SI 3056/15	2015	Food	Chicken carcass	Salmon_Fels_1, Salmon_SPN3UB
SI 4764/15	2015	Environment	Cleaning wipe	Gifsy_1, Entero_BP_4795, Salmon_118970_sal3, Salmon_SP_004
SI 5391/15	2015	Environment	Disposable shoe cover	Stx2_c_1717, Salmon_SW9
SI 5837/15	2015	Environment	Disposable shoe cover	Salmon_Fels_1, Salmon_SW9, Salmon_vB_SosS_Oslo
SI 5853/15	2015	Environment	Disposable shoe cover	Gifsy_1, Entero_BP_4795, Salmon_SW9
SI 5859/15	2015	Environment	Disposable shoe cover	Gifsy_1, Entero_BP_4795, Salmon_SW9
SI 5911/15	2015	Environment	Cleaning wipe	Gifsy_1, Entero_BP_4795, Salmon_118970_sal3
SI 5912/15	2015	Environment	Cleaning wipe	Gifsy_1, Entero_BP_4795, Salmon_118970_sal3
SI 5915/15	2015	Environment	Cleaning wipe	Gifsy_1, Entero_BP_4795, Salmon_118970_sal3, Entero_fiAA91_ss
SI 5923/15	2015	Environment	Cleaning wipe	Salmon_Fels_1, Entero_BP_4795, Salmon_118970_sal3
SI 220/16	2016	Environment	Cleaning wipe	Salmon_Fels_1, Salmon_118970_sal3, Stx2_c_1717
SI 3687/16	2016	Food	Chicken carcass	Gifsy_1, Entero_BP_4795, Salmon_118970_sal3
SI 4447/16	2016	Food	Pork sausage	Gifsy_1, Entero_BP_4795, Salmon_118970_sal3
SI 5946/16	2016	Food	Pork rib	Salmon_Fels_1, Salmon_118970_sal3
SI 6987/16	2016	Human	Human feces	Salmon_Fels_1
SI 7876/16	2016	Human	Human feces	Gifsy_1, Entero_BP_4795, Salmon_118970_sal3, Yersin_L_413C

Strain	Year	Isolation source	Isolation material	Prophages
SI 11/17	2017	Environment	Dragging swab	Salmon_Fels_1
SI 23/17	2017	Environment	Dragging swab	Salmon_Fels_1
SI 238/17	2017	Environment	Dragging swab	Gifsy_1
SI 872/17	2017	Food	Chicken carcass	Salmon_Fels_1, Yersin_L_413C
SI 1171/17	2017	Environment	Soil	-
SI 1256/17	2017	Environment	Soil	-
SI 2580/17	2017	Human	Human feces	Stx2_c_1717
SI 2953/17	2017	Human	Human fecal swab	Salmon_Fels_1, Yersin_L_413C
SI 2954/17	2017	Human	Human fecal swab	Salmon_Fels_1, Yersin_L_413C
SI 3380/17	2017	Human	Human fecal swab	Salmon_Fels_1, Yersin_L_413C
SI 3877/17	2017	Food	Chicken wings	Salmon_Fels_1, Escher_pro483
SI 3906/17	2017	Environment	Sieve residue	Salmon_Fels_1, Salmon_118970_sal3
SI 4065/17	2017	Human	Human feces	Salmon_Fels_1, Entero_BP_4795, Salmon_118970_sal3
SI 4067/17	2017	Human	Human feces	Gifsy_1, Entero_BP_4795, Salmon_118970_sal3
SI 4069/17	2017	Human	Human blood	Salmon_Fels_1
SI 52/18	2018	Food	Chicken carcass	Salmon_Fels_1, Yersin_L_413C
SI 331/18	2018	Human	Human fecal swab	Salmon_Fels_1
SI 623/18	2018	Human	Human feces	Yersin_L_413C, Entero_P4
SI 661/18	2018	Human	Human feces	Salmon_Fels_1
SI 942/18	2018	Human	Human fecal swab	Salmon_Fels_1, Entero_BP_4795, Salmon_118970_sal3
SI 1634/18	2018	Food	Yellowtail amberjack fish meat	Gifsy_1, Yersin_L_413C, Entero_P4
SI 2676/18	2018	Animal	Avian reproductive matrix	Gifsy_1, Escher_pro483
