# Supplementary material for: Comparative genomics reveals high genetic similarity among strains of Salmonella enterica serovar Infantis isolated from multiple sources in Brazil
Source: PeerJ. 2024 May 20;12:e17306. doi: 10.7717/peerj.17306 (PMC11114117; doi:10.7717/peerj.17306)
Supplement: Supplemental Information 1 — Sources: food (red), environmental (green), human (blue), animal (yellow), animal feed (orange). Reference genomes (black): Salmonella Infantis SINFA and Escherichia coli K-12. The profiles of antimicrobial resistance genes informed were described previously in (Vilela et al., 2022a) (Journal of Applied Microbiology 132:3327-3342 DOI: 10.1111/jam.15430) and (Vilela et al., 2022b) (PLOS ONE 17:e0277979 DOI: 10.1371/journal.pone.0277979). [file peerj-12-17306-s001.pdf]

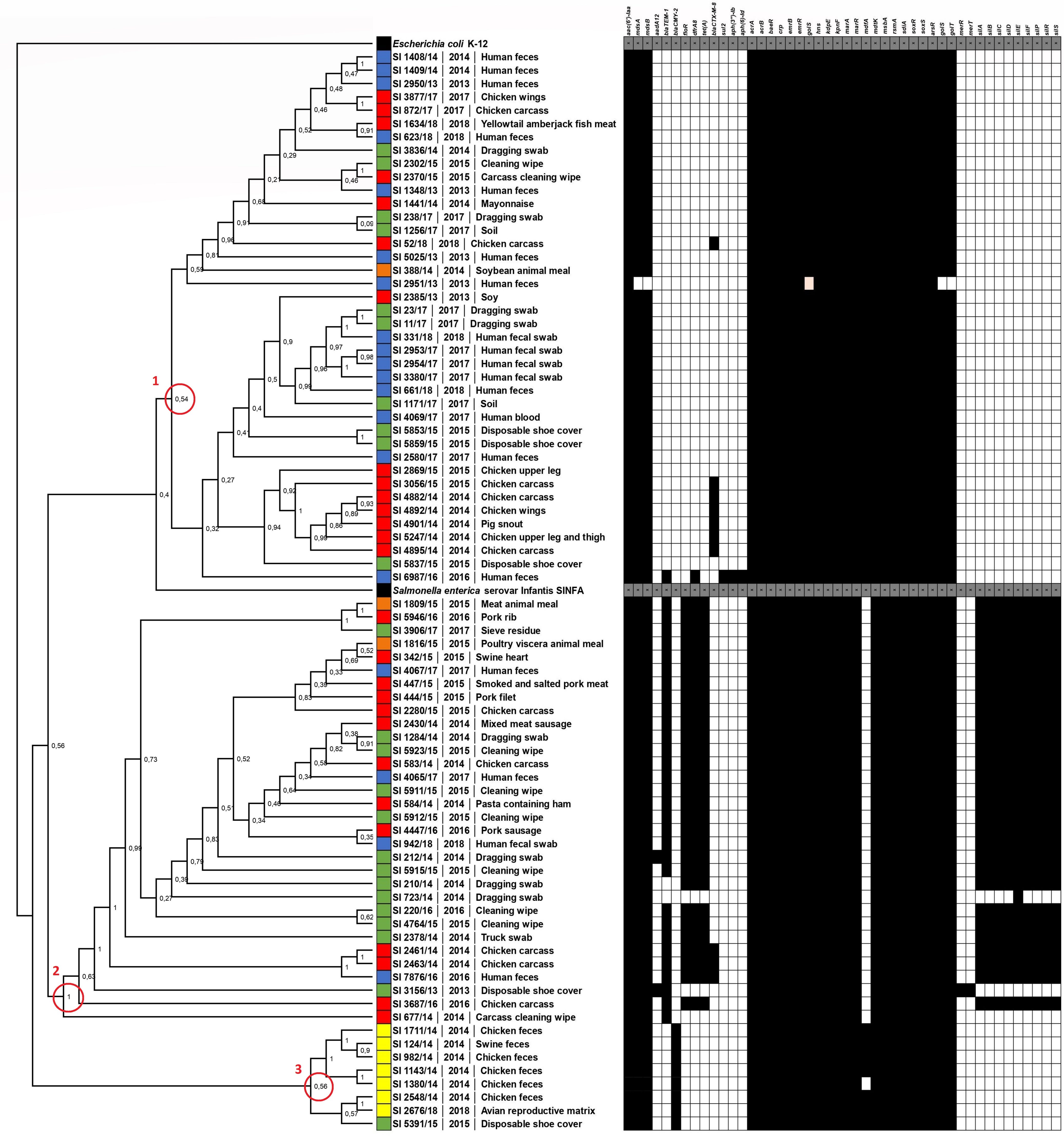

aac(6')-Iaa
mdsA
mdsB
aadA12
blaTEM-1
blaCMY-2
floR
dfrA8
tet(A)
blaCTX-M-8
sul2
aph(3'')-Ib
aph(6)-Id
acrA
acrB
baeR
crp
emrB
emrR
golS
hns
kdpE
kpnF
marA
marR
mdfA
mdtK
msbA
rsmA
sdiA
soxR
soxS
arsR
golS
golT
merR
merT
silA
silB
silC
silD
silE
silF
silP
silR
silS
1
2
3
Escherichia coli K-12
SI 1408/14 | 2014 | Human feces
SI 1409/14 | 2014 | Human feces
SI 2950/13 | 2013 | Human feces
SI 3877/17 | 2017 | Chicken wings
SI 872/17 | 2017 | Chicken carcass
SI 1634/18 | 2018 | Yellowtail amberjack fish meat
SI 623/18 | 2018 | Human feces
SI 3836/14 | 2014 | Dragging swab
SI 2302/15 | 2015 | Cleaning wipe
SI 2370/15 | 2015 | Carcass cleaning wipe
SI 1348/13 | 2013 | Human feces
SI 1441/14 | 2014 | Mayonnaise
SI 238/17 | 2017 | Dragging swab
SI 1256/17 | 2017 | Soil
SI 52/18 | 2018 | Chicken carcass
SI 5025/13 | 2013 | Human feces
SI 388/14 | 2014 | Soybean animal meal
SI 2951/13 | 2013 | Human feces
SI 2385/13 | 2013 | Soy
SI 23/17 | 2017 | Dragging swab
SI 11/17 | 2017 | Dragging swab
SI 331/18 | 2018 | Human fecal swab
SI 2953/17 | 2017 | Human fecal swab
SI 2954/17 | 2017 | Human fecal swab
SI 3380/17 | 2017 | Human fecal swab
SI 661/18 | 2018 | Human feces
SI 1171/17 | 2017 | Soil
SI 4069/17 | 2017 | Human blood
SI 5853/15 | 2015 | Disposable shoe cover
SI 5859/15 | 2015 | Disposable shoe cover
SI 2580/17 | 2017 | Human feces
SI 2869/15 | 2015 | Chicken upper leg
SI 3056/15 | 2015 | Chicken carcass
SI 4882/14 | 2014 | Chicken carcass
SI 4892/14 | 2014 | Chicken wings
SI 4901/14 | 2014 | Pig snout
SI 5247/14 | 2014 | Chicken upper leg and thigh
SI 4895/14 | 2014 | Chicken carcass
SI 5837/15 | 2015 | Disposable shoe cover
SI 6987/16 | 2016 | Human feces
Salmonella enterica serovar Infantis SINFA
SI 1809/15 | 2015 | Meat animal meal
SI 5946/16 | 2016 | Pork rib
SI 3906/17 | 2017 | Sieve residue
SI 1816/15 | 2015 | Poultry viscera animal meal
SI 342/15 | 2015 | Swine heart
SI 4067/17 | 2017 | Human feces
SI 447/15 | 2015 | Smoked and salted pork meat
SI 444/15 | 2015 | Pork filet
SI 2280/15 | 2015 | Chicken carcass
SI 2430/14 | 2014 | Mixed meat sausage
SI 1284/14 | 2014 | Dragging swab
SI 5923/15 | 2015 | Cleaning wipe
SI 583/14 | 2014 | Chicken carcass
SI 4065/17 | 2017 | Human feces
SI 5911/15 | 2015 | Cleaning wipe
SI 584/14 | 2014 | Pasta containing ham
SI 5912/15 | 2015 | Cleaning wipe
SI 4447/16 | 2016 | Pork sausage
SI 942/18 | 2018 | Human fecal swab
SI 212/14 | 2014 | Dragging swab
SI 5915/15 | 2015 | Cleaning wipe
SI 210/14 | 2014 | Dragging swab
SI 723/14 | 2014 | Dragging swab
SI 220/16 | 2016 | Cleaning wipe
SI 4764/15 | 2015 | Cleaning wipe
SI 2378/14 | 2014 | Truck swab
SI 2461/14 | 2014 | Chicken carcass
SI 2463/14 | 2014 | Chicken carcass
SI 7876/16 | 2016 | Human feces
SI 3156/13 | 2013 | Disposable shoe cover
SI 3687/16 | 2016 | Chicken carcass
SI 677/14 | 2014 | Carcass cleaning wipe
SI 1711/14 | 2014 | Chicken feces
SI 124/14 | 2014 | Swine feces
SI 982/14 | 2014 | Chicken feces
SI 1143/14 | 2014 | Chicken feces
SI 1380/14 | 2014 | Chicken feces
SI 2548/14 | 2014 | Chicken feces
SI 2676/18 | 2018 | Avian reproductive matrix
SI 5391/15 | 2015 | Disposable shoe cover
